# The Evolution of a Specialized, Highly Virulent Fish Pathogen through Gene Loss and Acquisition of Host-Specific Survival Mechanisms

**DOI:** 10.1128/aem.00222-22

**Published:** 2022-07-11

**Authors:** Laura Baseggio, Oleksandra Silayeva, Jan Engelstädter, Andrew C. Barnes

**Affiliations:** a The University of Queensland, School of Biological Sciences, Brisbane, Queensland, Australia; Norwegian University of Life Sciences

**Keywords:** *Photobacterium damselae*, host adaptation, horizontal gene transfer, vector-borne pathogen, pangenome, aquaculture, marine microbiology

## Abstract

Photobacterium damselae comprises two subspecies, P. damselae subsp. damselae and P. damselae subsp. *piscicida*, that contrast remarkably despite their taxonomic relationship. The former is opportunistic and free-living but can cause disease in compromised individuals from a broad diversity of taxa, while the latter is a highly specialized, primary fish pathogen. Here, we employ new closed curated genome assemblies from Australia to estimate the global phylogenetic structure of the species P. damselae. We identify genes responsible for the shift from an opportunist to a host-adapted fish pathogen, potentially via an arthropod vector as fish-to-fish transmission was not achieved in repeated cohabitation challenges despite high virulence for *Seriola lalandi*. Acquisition of ShdA adhesin and of thiol peroxidase may have allowed the environmental, generalist ancestor to colonize zooplankton and to occasionally enter in fish host sentinel cells. As dependence on the host has increased, P. damselae has lost nonessential genes, such as those related to nitrite and sulfite reduction, urea degradation, a type 6 secretion system (T6SS) and several toxin-antitoxin (TA) systems. Similar to the evolution of Yersinia pestis, the loss of urease may be the crucial event that allowed the pathogen to stably colonize zooplankton vectors. Acquisition of host-specific genes, such as those required to form a sialic acid capsule, was likely necessary for the emergent P. damselae subsp. *piscicida* to become a highly specialized, facultative intracellular fish pathogen. Processes that have shaped P. damselae subsp. *piscicida* from subsp. damselae are similar to those underlying evolution of Yersinia pestis from Y. pseudotuberculosis.

**IMPORTANCE** Photobacterium damselae subsp. damselae is a ubiquitous marine bacterium and opportunistic pathogen of compromised hosts of diverse taxa. In contrast, its sister subspecies P. damselae subsp. *piscicida* (*Pdp*) is highly virulent in fish. *Pdp* has evolved from a single subclade of *Pdd* through gene loss and acquisition. We show that fish-to-fish transmission does not occur in repeated infection models in the primary host*, Seriola lalandi,* and present genomic evidence for vector-borne transmission, potentially via zooplankton. The broad genomic changes from generalist *Pdd* to specialist *Pdp* parallel those of the environmental opportunist Yersinia pseudotuberculosis to vector-borne plague bacterium Y. pestis and demonstrate that evolutionary processes in bacterial pathogens are universal between the terrestrial and marine biosphere.

## INTRODUCTION

Lethal, host-specialized, obligate pathogens have often evolved from a less pathogenic opportunistic relative (1). A common feature in emerging, host-restricted pathogens is a reduced genome size and higher degree of pseudogenisation compared to the more generalist relatives (2). For example, obligate pathogens Mycobacterium tuberculosis and M. leprae have a substantially smaller genome compared to the free-living opportunist M. marinum (3).

Acquisition and loss of genes are the key mechanisms in bacterial evolution (4). As the host range of a pathogen narrows, its degree of dependence on the host increases. As the pathogen can rely on its host to provide many necessary metabolites, the biosynthetic pathways it no longer needs can be lost (5, 6). This leads to a gradual reduction in genome size and consequently in the energy required for replication of the chromosome set. Yet, novel genes may be required to extract the metabolites from the host and withstand its immune response. Plasmids are key vehicles for gene mobilization within and between species (7) and underpin the emergence of some of today’s most threatening pathogens, for example by promoting the transmission of virulence factors (8) and antibiotic resistance genes (9). Extensive research has focused on Yersinia pestis, the causative agent of human plague. Three plague pandemics have been attributed to this pathogen in the last 2000 years (10), with major social, political and economic consequences on populations. Evidence suggests that current strains of the highly specialized vector-borne pathogen Y. pestis evolved from a less pathogenic lineage present at least 5000 years ago (11). This lineage, in turn, seems to have diverged between 28000 and 54000 years ago from the fecal-orally transmitted generalist gastrointestinal pathogen Yersinia pseudotuberculosis (11). Relatively few steps of gene acquisition and gene loss were needed to allow Y. pseudotuberculosis to become the modern Y. pestis, with horizontally acquired plasmids providing two key events in this shift (12–14). The plasmids pPCP1 and pMT1, respectively, carrying the plasminogen activator Pla and the phospholipase D *ymt*, allowed Y. pestis to cause systemic infections and survival in the flea midgut. However, loss of gene function for either mutation or deletion also played a fundamental role in increasing Y. pestis biofilm formation ability and its transmissibility in the flea vector. While pseudogenisation of two phosphodiesterases (15) and mutation in a β-*N*-acetyl glucosaminidase (16) allowed stable, consistent colonization and obstruction of the flea foregut valve, the loss of urease activity due to mutation in *ureD* prevented intoxication of the flea host, preserving the host as a mobile disease vector (17). During this gradual adaptation to the new host landscapes, Y. pestis also started to increasingly rely on its hosts for essential resources, resulting in a relaxed selective pressure on genes involved in those biosynthetic or acquisitional pathways.

Photobacterium damselae is a multichromosome member of the family *Vibrionaceae* that comprises two remarkably different subspecies (18). While P. damselae subspecies *damselae* (Pdd) is a generalist, free-living opportunist, the subspecies P. damselae subspecies *piscicida* (Pdp) is a highly adapted, fish-restricted primary pathogen. Formerly known as Vibrio damsela and *Pasteurella piscicida,* respectively, Pdd and Pdp were subsequently reclassified based on identical 16S rRNA sequences and their degree of DNA hybridization (19). Despite their taxonomical classification, Pdd and Pdp are very different. Pdd genomes vary considerably among strains (20), resulting in high genetic heterogeneity not only at a local level, i.e., presence/absence of strain-specific siderophores or secretion systems, but also differing in plasmid content (18). In Pdd, the most virulent strains carry four toxin-encoding genes: two on chromosome 1, the phospholipase toxin PlpV and the pore forming toxin phobalysin C (PhlyC), and two on the pPHDD1 plasmid, damselysin (Dly) and the pore forming toxin phobalysin P (PhlyP) (21–23). However, plasmid-free strains are also frequently isolated from infected individuals (24). So far, Pdd infections have been recorded in a variety of animals, including fish, cetaceans, crustaceans, and humans. However, Pdd seems to be fatal to stressed animals (25) or individuals with preexisting medical conditions or whose skin integrity, the body’s first line of defense, was compromised (26, 27). In contrast, Pdp strains are genetically homogeneous when comparing chromosomal DNA content. Pdp virulence relies on two plasmid-borne toxins: AIP56 on plasmid pPHDP10 (28) and the siderophore piscibactin on plasmid pPHDP70 (29). However, it was not until 1990, when Pdp mortality events were first recorded in Europe (30), that these two plasmids were identified together (31, 32). Other potential virulence factors have been proposed, but no functional studies have been conducted to date to assess their role (30, 33–35). Similar to other host-adapted pathogens, the Pdp genome is reduced in size and functional gene content compared to Pdd (33). An extensive proliferation of insertion sequence elements has led to a generalized pseudogenization, reduced metabolic plasticity and genomic streamlining (36). Importantly, Pdp has also been shown to thrive intracellularly in various types of host cells (37–39), and by expressing the plasmid-encoded toxin AIP56 it can induce apoptosis in fish sentinel cells (40). However, it is still unclear how Pdp persists within the highly oxidative environment of macrophages. Furthermore, little is known to date about Pdp route of transmission and the existence of potential reservoirs or carriers (33, 41).

Here, we investigated the genomic changes underpinning the evolution of a highly adapted primary fish pathogen, Pdp, from an opportunistic generalist, Pdd. We identified key genes that might have allowed the transition of Pdp from colonization of the extracellular milieu to the invasion of several types of cells, including macrophages, and survival of the oxidative burst within the latter. Finally, we provided evidence that the mechanisms of evolution usually associated with terrestrial bacterial pathogens also apply to marine bacteria.

## RESULTS AND DISCUSSION

P. damselae is a complex multichromosome aquatic bacterial species comprising two subspecies, P. damselae subsp. damselae and P. damselae subsp. *piscicida.* Although both important threats in global marine aquaculture, these two microorganisms exhibit very different lifestyles and therefore represent a good model for the investigation of pathogenicity evolution and host adaptation.

### Phylogenetics.

**FIG 1:**
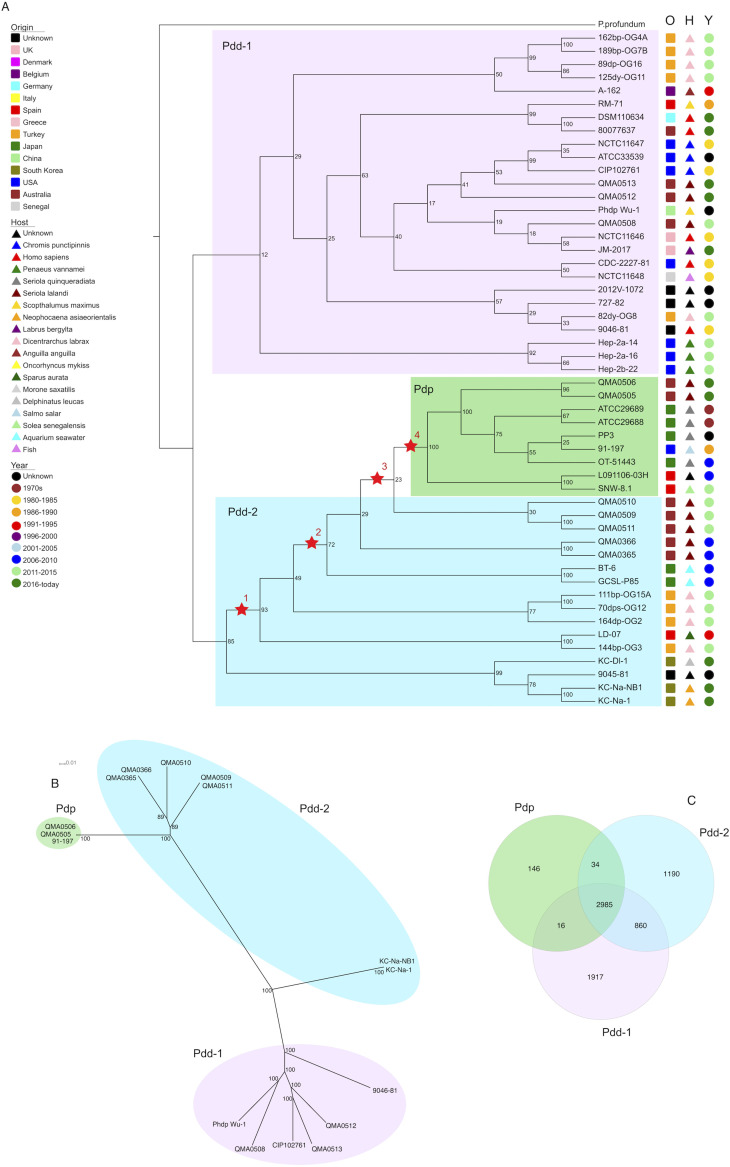
Maximum Likelihood Phylogeny of Photobacterium damselae (A) Complete phylogenetic cladogram of Photobacterium damselae. The origin (O), host (H) and year of isolation (Y) are indicated and Photobacterium profundum is used to root the cladogram. The three clades are highlighted in different colors and the four stars indicate key changes in the genomes: (i) acquisition of disulfide bond formation protein B and thiol peroxidase; (ii) acquisition of glutathione *S*-transferase family protein; (iii) acquisition of Ig-like domain-containing protein with adhesin ShdA domain and serine/threonine protein kinase; (iv) partial AlpA family phage integration, Stealth CR1 domain-containing protein and capsular operon. (B) Phylogram of Pdp and Pdd genomes fully assembled and closed. Bootstrap values are indicated next to the nodes and colors reflect the three clades from panel (A. C) Venn diagram representing the number of genes that are always present in all species of one clade but nowhere else (146 in Pdp, 1917 in Pdd-1, 1190 in Pdd-2), in common between genes that are always present in each clade but not elsewhere (860 between Pdd-1 and Pdd-2, 16 between Pdd-1 and Pdp, 34 between Pdd-2 and Pdp) and the 2985 P. damselae core genes.

Phylogenetic analysis based on 7876 nonrecombinant core genome single nucleotide polymorphisms (SNPs) of 62 P. damselae genome assemblies from different continents, including Europe, Asia, America, Africa and Oceania (Table 1), revealed the presence of three major clades, here denoted Pdd-1, Pdd-2 and Pdp (Fig. 1). These core-genome-derived clades are broadly similar to those identified by alignment of the *toxR* gene (42) (where the strains AS-15-3942-7, AS-15-3942-8, AS-15-3942-9, AS-16-0963-1 and AS-16-0963-3 are QMA0509, QMA0510, QMA0511, QMA0512 and QMA0513, respectively), but whole-genome data and the higher number of strains included here allowed a more comprehensive representation and higher resolution of P. damselae subsp. phylogeny.

**TABLE 1:** Photobacterium damselae subsp. damselae (white background) and subsp. *piscicida* (gray background) genome assemblies currently available on NCBI^*a*^

Strain (BioSample)	yr	Level^*b*^	GenBank accession no.	Assembly method	Coverage (x)	Technology
KC-Na-1 (SAMN06909323)	2017	****	GCA_002142615.1	HGAP3 v.Apr-2017	218	PacBio; Illumina HiSeq
Phdp Wu-1 (SAMN06075355)	2018	****	GCA_003130755.1	SMRT portal v.2016-11-23	100	PacBio
91-197 (SAMD00078763)	2017	****	GCA_002356235.1	HGAP v.3	343.5	PacBioRSII
9046-81 (SAMN12648307)	2019	****	GCA_009763125.1	HGAP v.3	217.8	PacBio
KC-Na-NB1 (SAMN10797285)	2019	****	GCA_004135105.1	HGAP v.Apr-2017	242	Illumina HiSeq; PacBio RSII
QMA0365 (SAMN16287395)	2020	****	CP090501- CP090503	Flye v.2.6; Unicycler v.0.4.8	90	Oxford Nanopore MinION; Illumina NextSeq
QMA0366 (SAMN16287396)	2020	****	CP090498 -CP090500	Flye v.2.6; Unicycler v.0.4.8	90	Oxford Nanopore MinION; Illumina NextSeq
QMA0505 (SAMN16091814)	2020	****	GCA_014775675.1	Flye v.2.6; Unicycler v.0.4.8	90	Oxford Nanopore MinION; Illumina NextSeq
QMA0506 (SAMN16091815)	2020	****	GCA_014775695.1	Flye v.2.6; Unicycler v.0.4.8	26	Oxford Nanopore MinION; Illumina NextSeq
QMA0508 (SAMN16287397)	2020	****	CP090495-CP090497	Flye v.2.6; Unicycler v.0.4.8	90	Oxford Nanopore MinION; Illumina NextSeq
QMA0509 (SAMN16287398)	2020	****	CP090493- CP090494	Flye v.2.6; Unicycler v.0.4.8	90	Oxford Nanopore MinION; Illumina NextSeq
QMA0510 (SAMN16287399)	2020	****	CP090489- CP090492	Flye v.2.6; Unicycler v.0.4.8	90	Oxford Nanopore MinION; Illumina NextSeq
QMA0511 (SAMN16287400)	2020	****	CP090487- CP090488	Flye v.2.6; Unicycler v.0.4.8	90	Oxford Nanopore MinION; Illumina NextSeq
QMA0512 (SAMN16287401)	2020	****	CP090485- CP090486	Flye v.2.6; Unicycler v.0.4.8	90	Oxford Nanopore MinION; Illumina NextSeq
QMA0513 (SAMN16481684)	2020	****	CP065041- CP065043	Flye v.2.6; Unicycler v.0.4.8	90	Oxford Nanopore MinION; Illumina NextSeq
2012V-1072 (SAMN10702680)	2019	***	GCA_009665375.1	HGAP v.2	228.6	PacBio
CIP 102761 (SAMN02393816)	2009	**	GCA_000176795.1	na	8	Sanger; 454
LD-07 (SAMN04531897)	2016	**	GCA_001707815.1	SPAdes v.3.6	100	Illumina MiSeq
RM-71 (SAMN04531896)	2016	**	GCA_001708035.1	SPAdes v.3.6	100	Illumina MiSeq
89dp-OG16 (SAMN11608958)	2019	**	GCA_005819885.1	SPAdes v.3.6	200	Illumina MiSeq
64bp-OG9 (SAMN11608778) Excluded^*1*^	2019	**	GCA_005819845.1	SPAdes v.3.6	200	Illumina MiSeq
70dps-OG12 (SAMN11608782)	2019	**	GCA_005819805.1	SPAdes v.3.6	200	Illumina MiSeq
111bp-OG15A (SAMN11608899)	2019	**	GCA_005819825.1	SPAdes v.3.6	200	Illumina MiSeq
144bp-OG3 (SAMN11608214)	2019	**	GCA_005819835.1	SPAdes v.3.6	200	Illumina MiSeq
DI21 (SAMN02471934) Excluded^*1*^	2015	**	GCA_000300355.4	Newbler v.2.6	60	454
L091106-03H (SAMN05450422)	2017	**	GCA_001715165.2	CABOT v.JAN-2015; SOAPdenovo v.JAN-2015; SSPACE v.JAN-2015	50	454
ATCC 33539 (SAMN07327718)	2018	*	GCA_003026815.1	SPAdes v.3.11.0	121	Illumina MiSeq
OT-51443 (SAMD00076069)	2017	*	GCA_002157645.1	HGAP v.3	343.5	PacBio RSII
NCTC11646 (SAMEA104210785)	2018	*	GCA_900454675.1	na	100	na
NCTC11648 (SAMEA104338370)	2018	*	GCA_900454595.1	na	100	PacBio RS
BT-6 (SAMN07327681)	2018	*	GCA_003025675.1	SPAdes v.3.11.0	97	Illumina MiSeq
JM-2017 (SAMN10748379)	2019	*	GCA_004794735.1	SPAdes v.3.12	11.4	Illumina MiSeq
164dp-OG2 (SAMN11607985)	2019	*	GCA_006297395.2	SPAdes v.3.6	60	Illumina MiSeq
A-162 (SAMN05178634)	2016	*	GCA_001729115.1	SPAdes v.3.6	100	Illumina MiSeq
Hep-2b-22 (SAMN08810358)	2019	*	GCA_006124915.1	MaSuRCA v.3.1.3	586	Illumina MiSeq
Hep-2a-14 (SAMN08810065)	2019	*	GCA_006124835.1	MaSuRCA v.3.1.3	595	Illumina HiSeq
Hep-2a-16 (SAMN08810066)	2019	*	GCA_006124795.1	MaSuRCA v.3.1.3	551	Illumina HiSeq
80077637 (SAMN12838464)	2019	*	GCA_008806825.1	SPAdes v.3.6	60	Illumina MiSeq
Hep-2a-11 (SAMN05195137) Excluded^*2*^	2018	*	GCA_003355625.1	Velvet v.1.2.10	80	Illumina MiSeq
940804-1/1 (SAMN08536167) Excluded^*1*^	2018	*	GCA_003004585.1	SPAdes v.3.6	100	Illumina MiSeq
CDC-2227-81 (SAMN12830090)	2019	*	GCA_008806795.1	SPAdes v.3.6	60	Illumina MiSeq
206328-2 (SAMN08536175) Excluded^*1*^	2018	*	GCA_003004595.1	SPAdes v.3.6	100	Illumina MiSeq
940804-1/2 (SAMN08536174) Excluded^*1*^	2018	*	GCA_003004505.1	SPAdes v.3.6	100	Illumina MiSeq
206317-1 (SAMN08536176) Excluded^*1*^	2018	*	GCA_003004605.1	SPAdes v.3.6	100	Illumina MiSeq
ATCC 29688 (SAMN07327717)	2018	*	GCA_003026775.1	SPAdes v.3.11.0	88	Illumina MiSeq
ATCC 29689 (SAMN07327716)	2018	*	GCA_003026245.1	SPAdes v.3.11.0	70	Illumina MiSeq
PP3 (SAMN11269591)	2019	*	GCA_004683985.1	Megahit v.1.0	100	Illumina MiSeq
SNW-8.1 (SAMN11269579)	2019	*	GCA_004684005.1	SPAdes v.3.6	50	Illumina MiSeq
MT1415 (SAMN11479479) Excluded^*1*^	2019	*	GCA_005048985.1	Megahit v.1.0	200	Illumina MiSeq
206352-6 (SAMN15312412) Excluded^*1*^	2020	*	GCA_013371045.1	SPAdes v.3.6	50	Illumina MiSeq
162bp-OG4A (SAMN15312464)	2020	*	GCA_013371545.1	SPAdes v.3.6	50	Illumina MiSeq
CDC-1421-81 (SAMN15366240) Excluded^*1*^	2020	*	GCA_013377605.1	Megahit v.1.0	50	Illumina MiSeq
82dy-OG8 (SAMN15313879)	2020	*	GCA_013377775.1	SPAdes v.3.6	50	Illumina MiSeq
189bp-OG7B (SAMN15312514)	2020	*	GCA_013377875.1	SPAdes v.3.6	50	Illumina MiSeq
125dy-OG11 (SAMN15659787)	2020	*	GCA_014042565.1	SPAdes v.3.6	25	Illumina MiSeq
727-82 (SAMN13500474)	2020	*	GCA_014525015.1	HGAP v.3	71.8	PacBio
9045-81 (SAMN12648252)	2020	*	GCA_014525275.1	HGAP v.3	101.8	PacBio
KC-Dl-1 (SAMN16393141)	2020	****	GCA_014930975.1	HGAP v. Oct-2020	282	Illumina HiSeq; PacBioRSII
DSM110634 (SAMN14467235)	2020	*	GCA_015602445.1	SPAdes v.3.14	75	Illumina NextSeq
MT-1590 (SAMD00109182) Excluded^*3*^	2020	*	GCA_016756435.1	HGAP v.3	343.5	PacBioRSII
NCTC11647 (SAMEA4362424)	2018	*	GCA_900454585.1	na	100	na
NCTC11648 (SAMEA104338370)	2018	*	GCA_900454595.1	na	100	na
NCTC11646 (SAMEA104210785)	2018	*	GCA_900454675.1	na	100	na

a Exclusion reasons: ^*1*^16S rRNA was not annotated in the original assemblies nor found by BLAST; ^*2*^the strain species is uncertain as Hep-2a-11 is identified as both P. damselae and Vibrio parahaemolyticus on NCBI; ^*3*^the assembly does not represent the whole genome of the strain.

b In ‘Level’, **** denotes the assembly levels as complete genome, *** chromosome, ** scaffold, and * contigs. (na, not available). Some genome assemblies were excluded from the analyses.

Within the Pdp clade, geographical endemism clearly separates European from Asian and Australian isolates, as previously described (33). The Pdp clade is nested within one of the Pdd groups, indicating that some Pdd strains are more closely related to Pdp than to other Pdd. The closest strains to Pdp are five Australian strains isolated from *Seriola lalandi* in 2015 (QMA0509, QMA0510, QMA0511, Western Australia) and 2010 (QMA0365 and QMA0366, South Australia). Within and between the two distinct Pdd clades, we found no evident driver of the clustering in terms of geographic origin, host species or year of isolation (Fig. 1), nor was there any temporal signal in the phylogeny when a root-to-tip regression of branch length against isolation date was conducted (Fig. S3 Supplemental material).

Similar to other *Vibrionaceae* (43), genes constituting the core genome of P. damselae are mainly located on chromosome 1. The second chromosome, which may have been originally a ‘megaplasmid’ (44), shows a higher degree of variability between strains (Fig. S4 Supplemental material). We found that the highly specialized fish pathogen Pdp has evolved from a subgroup of Pdd and we identified genes that may have contributed to the host-adaptation process.

### Gene presence/absence.

The core genome of Photobacterium damselae based on 62 strains of diverse origin comprises 2985 genes. The function of these genes is primarily linked to essential metabolism, replication, and growth (Fig. 1C), which were retained in Pdp despite general reduction of the genome (Fig. S5 Supplemental material). The transition from Pdd and Pdp has been gradual (Fig. 1A) and to better understand the genomic and phenotypic changes that have occurred, we focused on each breakpoint from Pdd-2 to Pdp and examined the differences between ancestors and descendents. At the first bifurcation (Fig. 1A, Node 1), separating the Korean isolates, two proteins were acquired. The first is a disulfide bond formation protein B with 98% coverage and 77.47% identity in Aliivibrio sifiae (WP_105055669.1). A thiol peroxidase (*tpx*) has also been acquired, but its origin is uncertain: the highest coverage and identity is with *tpx* from the newly characterized species *Spartinivicinus ruber* (WP_163835251.1) and *Endozoicomonas* sp. SM1973 (WP_180567802.1) (99% coverage with 65.85% and 65.24% identity). The former has been recently isolated and solely described as a red pigment-producing species (45). The latter genus is a globally distributed symbiotic bacterium of a wide range of animals (sponges, mollusks, tubeworms, fish) (46). However, the Australian and Japanese Pdd strains and all Pdp strains (Fig. 1A, Node 2) more likely acquired a glutathione *S*-transferase family protein from Vibrio campbellii (99% identity and 78.88% coverage), despite the smaller coverage value. Finally, only in QMA0509 and QMA0511 (Fig. 1A, Node 3), an Ig-like domain-containing protein that contains a fibronectin-binding autotransporter adhesin ShdA domain (Vibrio harveyi 98% coverage and 31.72% identity) and a serine/threonine protein kinase (*Vibrio* sp. 070316B with 80% coverage and 46.41% identity) were acquired. The genes that are only present in Pdp (Fig. 1A, Node 4) are related to capsule formation, extracellular polysaccharide biosynthesis, and to immune system evasion (Table 2). All Pdp strains also have a conserved integrated phage region that encodes two AlpA family phage regulatory proteins, a toprim domain-containing protein and two hypothetical proteins.

**TABLE 2:** Genes present in Pdp isolates only

Function	Genes	BLAST (%coverage, %identity)
Capsule formation and extracellular polysaccharides biosynthesis	Polysaccharide biosynthesis/export family protein	Vibrio owensii (66, 99)
	Low mol wt phosphotyrosine protein phosphatase	Vibrio cyclitrophicus (54, 98)
	Polysaccharide biosynthesis tyrosine autokinase	Vibrio hyugaensis (65, 97)
	Oligosaccharide flippase family protein	Photobacterium leiognathi (60, 99)
	Glycosyltransferase family 25 protein	Vibrio furnissii (64, 99)
	EpsG family protein	Aeromonas caviae (31, 96)
	CMP-N-acetylneuraminate-beta-galactosamide-alpha-2,3-sialyltransferase	Vibrio hyugaensis (81, 99)
Immune system evasion	Stealth CR1 domain-containing protein	Vibrio jasicida (60, 99)
Partial integrated phage	AlpA family phage regulatory proteins	Vibrio parahaemolyticus (74, 96)
	Toprim domain-containing protein	Photobacterium leiognathi (58, 99)
	Hypothetical protein	Vibrio parahaemolyticus (40, 98)
	Hypothetical protein	Vibrio vulnificus (98)

Pdd-2 isolates and Pdp share several genes that encode proteins related to oxidative stress resistance, i.e., disulfide bond formation protein B, thiol peroxidase, glutathione *S*-transferase family protein. These genes may have been crucial in Pdd-2 isolates allowing them to successfully survive oxidative burst inside macrophages. In Salmonella enterica, the *tpx* gene has been demonstrated to provide protection against hydrogen peroxide and to facilitate intracellular survival (47). The copy number of the gene further supports this hypothesis. While Pdp has only one copy of *txp*, the Pdd-2 isolates carry two different copies of *tpx*. This could indicate that originally a higher gene dosage was needed for adequate support against oxidative stress during occasional invasions of sentinel cells, while Pdp, being a highly specialized, facultatively intracellular fish pathogen had to evolve more sophisticated defenses. Indeed, Pdd-2 strains were more resistant to H_2_O_2_ (Fig. S6 Supplemental material) compared to Pdp and the Pdd-1 strains. At the final step in the phylogenetic transition from Pdd to Pdp, the majority of acquired genes provide protection from the fish immune system. First, capsule and extracellular polysaccharide biosynthesis regions differ considerably, with Pdp having a distinct sialic acid capsular operon absent in Pdd. Importantly, the CMP-N-acetylneuraminate-beta-galactosamide-alpha-2,3-sialyltransferase is unique to Pdp isolates and it may be one of the key genes that allowed Pdp evasion from the fish immune system. This enzyme is involved in the formation of polysialic capsules whose negative charges impede the activation of the host humoral immune defenses (48) and displays antiphagocytic activity (49). Many successful pathogens, including Pasteurella haemolytica A2 (50), Pasteurella multocida (51), Neisseria meningitidis (52), and Haemophilus influenzae (53) express polysialic acid capsules. Differences in capsular structure appears to be fundamental for enhanced virulence in Pdp. Importantly, in all Pdp strains an integrated partial phage on chromosome 1 appears to be able to control the expression of the capsule. In particular, an AlpA family phage regulatory protein is present in double copy (IC627_14400 and IC627_14405) and has been demonstrated to suppress capsule overproduction and UV sensitivity in delta Lon protease mutants (54). Indeed, UV light causes oxidative stress damage (55), a condition that Pdp needs to overcome once inside fish macrophages. In addition to sialic acid as a concealment strategy, a Stealth CR1 domain-containing protein is uniquely found in Pdp (IC627_20775). These proteins are considered to confer protection against the host innate immune defenses, consequently Stealth proteins are found mainly in pathogens, including Neisseria meningitidis, Streptococcus pneumoniae, Mycobacterium leprae, and M. tuberculosis (56).

Pdp strains, however, have lost more genes than the well-reported urease and hemolysins genes (18). All Pdd isolates contain a Type VI secretion system (T6SS) gene cluster, while in Pdp only *tssI*, encoding type VI secretion system tip protein VgrG, remained in triple copy. Genes related to iron metabolism like EfeBOU, SufBC and *HmuU* are absent in Pdp, together with AsrABC for anaerobic sulfite reduction, NirCD for nitrite reduction and the maltose uptake system MalEFG. Several type II toxin-antitoxin systems (HipAB, Phd/YefM family antitoxin, type II toxin-antitoxin system prevent-host-death family antitoxin, antitoxin HigA1, Txe/YoeB family addiction module toxin) are found in all Pdd strains, while only the HigB and type II toxin-antitoxin system RelE/ParE family toxin are shared between the two subspecies. The loss of multiple metabolic pathways clearly reflects the host-adaptation process that Pdp has undergone, while the free-living Pdd must be able to withstand a variety of changing environmental stressors. In type II toxin-antitoxin (TA) systems, toxins inhibit the growth and may cause cell death of neighboring organisms that do not encode the respective antitoxin protein (57). The T6SS is a complex, contractile machinery that delivers lethal effector proteins into adjacent cells (58, 59). Both mechanisms are beneficial to free-living microorganisms like Pdd that live in mixed communities and biofilms.

### Capsular and lipopolysaccharide O-antigen biosynthesis operons.

**FIG 2:**
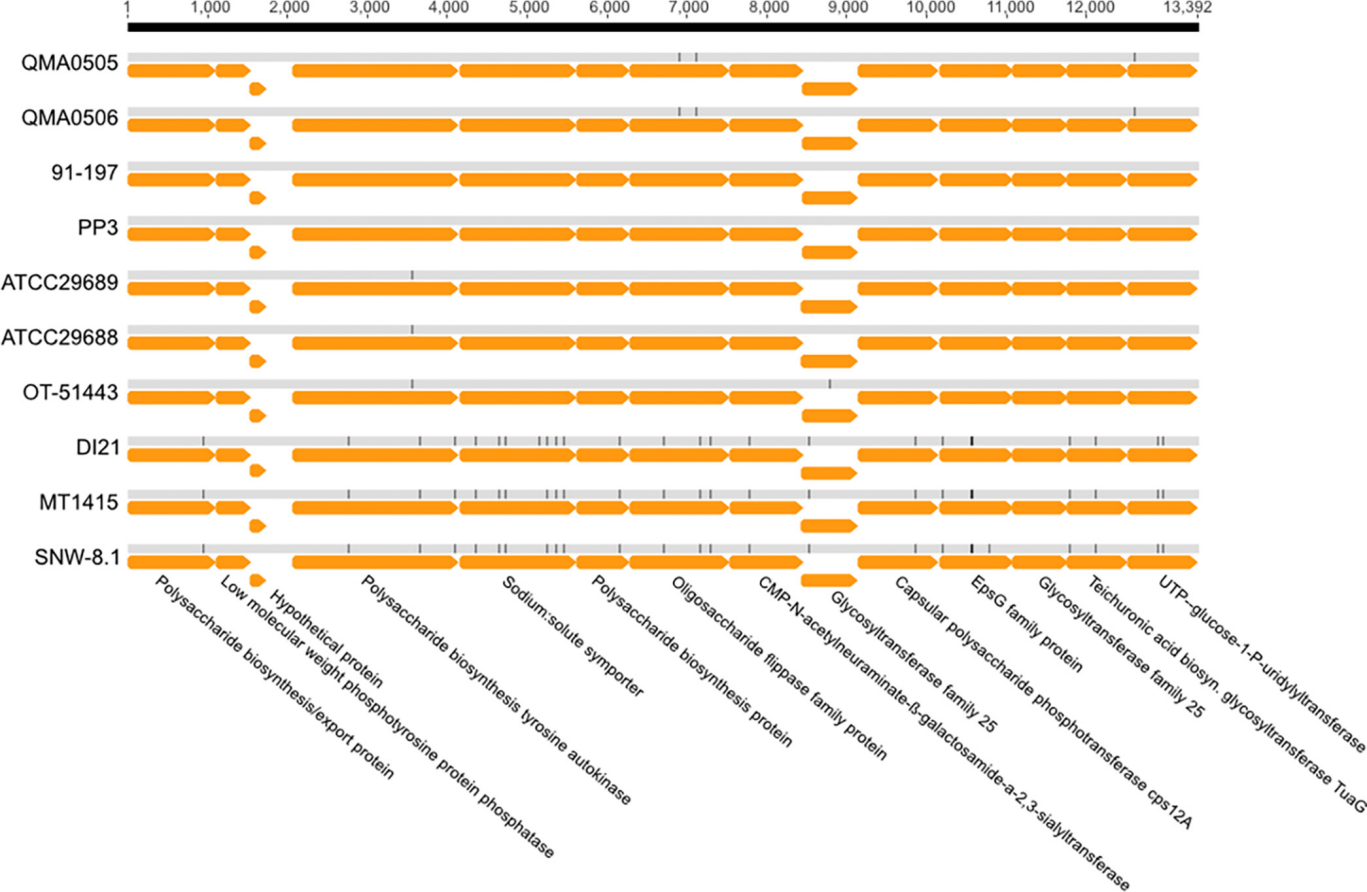
Alignment of Pdp capsular operon in Pdp strains. Orange arrows indicate coding sequences (CDSs) and black vertical dashes represent single nucleotide polymorphisms (SNPs).

We report here, for the first time, the highly conserved Pdp capsular operon located on chromosome 2. In 13392 bp, the number of single nucleotide polymorphisms (SNPs) is 25, with the majority distinguishing European strains (DI21, MT1415, and SNW-8.1) from the other isolates (Fig. 2). In contrast, Pdd strains exhibit a complex region on chromosome 1 that encodes both lipopolysaccharide (LPS) O-antigen and capsule production. Australian Pdd strains have high variability in both gene composition and gene sequences, as shown previously for Danish strains isolated in 1994 and 2006 (25).

### Flagellar operon regions.

In both subspecies of P. damselae a putative flagellar biosynthesis cluster is located on chromosome 1 (Fig. S7 Supplemental material). Compared, these operons contain major differences. In Pdp, insertion sequence elements (ISEs) have inactivated several genes and, importantly, the transcription regulators controlling flagellar expression. The RNA polymerase sigma factor FliA and the sigma-54-dependent Fis family transcriptional regulator FleQ, as well as the flagellum site-determining protein YlxH, the chemotaxis protein CheV and the flagella assembly protein FlgT have been fragmented by ISEs. Other pseudogenes include the flagellar sensor histidine kinase FleS, the flagellar hook protein FlgE and a hypothetical protein with the FlgO protein domain. Only partial genes encoding the flagellin lysine-N-methylase and the flagellar hook-associated protein FlgK and flagellar filament capping protein FliD remain, while the gene for the flagellar hook-associated protein FlgL has been entirely lost. Despite facilitating adhesion to host tissues, flagellin is also a very potent Pathogen Associated Molecular Pattern (PAMP) that initiates innate immune responses in the host. Flagellin has been shown to activate proinflammatory cytokines, acute phase proteins, and antimicrobial peptides in rainbow trout (Oncorhynchus mykiss) (60, 61), Atlantic salmon (*Salmo salar*) (62) and gilthead seabream (Sparus aurata) (61). Repression of the flagellar machinery is also found in Salmonella enterica Serovar Dublin isolates (63) recovered from invasive cases of salmonellosis, indicating that aflagellated strains can rapidly spread systemically by avoiding the initiation of the host immune response. Conversely, the flagellar operon in Pdd strains is functional as it is involved in many key processes in free-living, environmental species, such as motility, biofilm formation and dispersal (64).

### Plasmids, horizontal gene transfer and adaptation.

Horizontal gene transfer (HGT) plays a major role in host adaptation. When acquired, many genes initially are expressed at low levels, providing no major effects in the recipient phenotype (7). Later, novel genes are fully expressed and retained if they confer a fitness advantage, otherwise unnecessary genes are lost. Plasmids mobilize sets of genes across individuals within the same but also between different and distantly related species, providing a growing genetic pool from which each strain can draw. P. damselae pangenome is currently almost 20 Mbp, encoding more than 7000 genes (excluding ISEs and transposases). However, when comparing the two subspecies, the Pdd pangenome is larger than that of Pdp, accounting for 4001 and 3181 genes, respectively, but this may be due to the number of genomes present in each group. A larger number of high-quality genome assemblies of Pdp is needed to better assess this difference. Many host-restricted pathogens, such as Yersinia pestis, Salmonella enterica serovar Typhi, and Mycobacterium leprae have undergone a similar process of genome reduction mediated by expansion of ISEs (2). These ISEs insert themselves into functional open reading frames (ORFs), fragmenting them and inactivating the genes. The flagellar operon in Pdp constitutes an important example of gene inactivation. Usually considered nonmotile for the absence of flagella, some Pdp strains have been reported to use pili for twitching motility (65). Pdp may have retained only pili as flagellin is a potent activator of the immune system. The flagellin gene in Pdp is shorter than those in Pdd and some components of the flagellar building machinery have been lost completely.

Plasmids are generally recognized as major drivers of HGT. The Pdd plasmidome varies extensively both in terms of quantity per isolate and type of plasmid. Recently isolated Pdp strains, in turn, have a more conserved set of plasmids, including the *aip56*-bearing pPHDP10 and the piscibactin-encoding pPHDP70. A virulence gene screen has shown that only 4 strains out of 79 isolated after 2002 lack piscibactin, and only some early isolates before 1995 do not carry the two plasmids (30). As 91–197 isolated in 1991 in the US lacks both plasmids, AIP56 and piscibactin are not part of Pdp core genome. This may indicate that key Pdp plasmids have become stable elements over time providing the tools for optimal virulence in fish. This pathogen can thrive both extra- and intracellularly in various cell types, including macrophages and neutrophils. Conversely, the generalist Pdd relies on a larger gene pool, broadening its ability to respond to environmental changes. This genetic variability poses a serious challenge to serotyping and vaccine development as cross-protection between strains may be difficult to achieve.

### Efficient transmission of Pdp relies on compromised mucosal layer of fish.

The results conclusively show the lack of efficient Pdp transmission through water and direct contact in Yellowtail Kingfish with intact skin/mucosal layer. Although, it transmits readily if the mucosal/skin layer is compromised. This was demonstrated in fish of different size, by different experimental approaches, and experimental environments.

**FIG 3:**
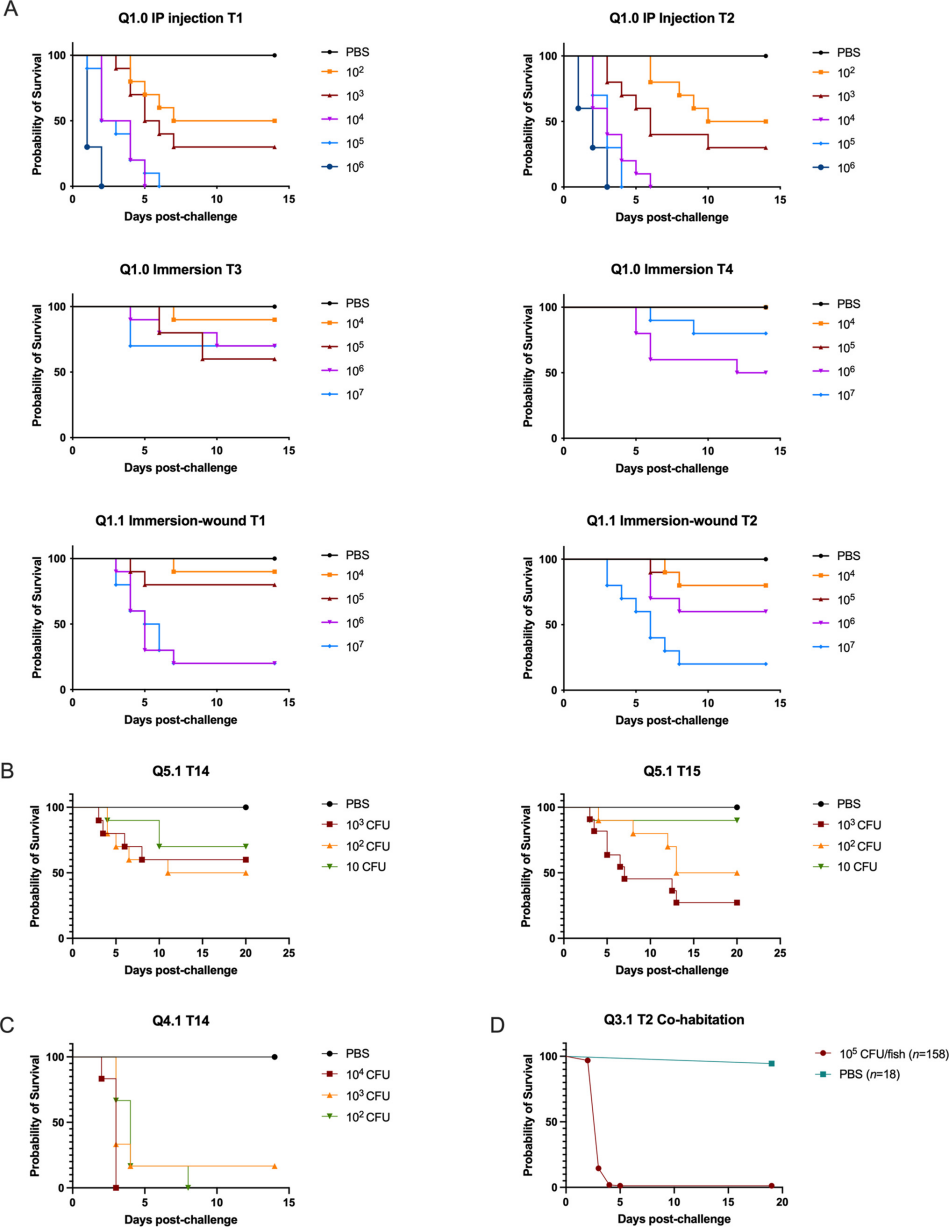
Infection transmission from challenge trials experiments conducted between 2019 and 2021. (A) Route of infection experiment Q1. Survival curves after challenge with different concentrations of Pdp QMA0506. Row 1 to 3 intraperitoneal injection, immersion, and immersion after wounding with two tank replicates each (Curve comparison significantly different *P* < 0.0001, Mantel-Cox log-rank test). (B) and (C) and (D) Prechallenge pathogen dose titration experiments in different cohorts of fish (Curve comparison significantly different *P* = 0.002, Mantel-Cox log-rank test). (D) Commercial vaccination challenge experiment Q3.1 in 120 g fish stocked at 26.4 kg/m^3^ with cohabiting unchallenged fish. Survival curves of challenged (10^5^ CFU of QMA0506 Pdp strain in 100 μL of PBS by IP injection) and unchallenged (IP injection of 100 μL of PBS) (Curve comparison significantly different *P* < 0.0001, Mantel-Cox log-rank test). 100% mortality was recorded in challenged fish after 3 days. 1 unchallenged fish out of 18 showed Pdp infection signs 6 days postchallenge. This fish was mishandled during transfer from anesthetic and developed a large hematoma on its head. In every other case, there was no transfer of infection to unchallenged fish cohabiting in the tanks regardless of size or genetic background of the cohort.

In the infection model/delivery route comparison experiment, the three experimental designs of the challenge of Yellowtail Kingfish (*Seriola lalandi*) with Pdp gave remarkably different results (Fig. 3), yet in all three designs all moribund animals were positive to Pdp isolation and PCR, and all surviving fish were negative. In IP injection challenge, a clear dose-dependent response was detected and the survival curves were similar between the two tanks, indicating a high level of replicability. In contrast, immersion challenge resulted in no consistent dose-dependent response. However, the immersion after wounding of the fish resulted in dose-dependent survival with the highest concentrations of Pdp leading to high levels of mortality. The relatively high survival of animals in the immersion challenge may indicate that Pdp primary route of infection is unlikely to happen through water. In fact, despite the greater intrinsic variability of an immersion challenge (as the number of pathogens infecting each individual cannot be accurately controlled and there may be small differences among the group), there is no trend suggesting that higher concentrations lead to higher mortality. Instead, it may be necessary for Pdp to be routed through a compromised mucosal layer (as in the immersion after wound challenge), suggesting possible blood-borne pathogenicity.

Lack of transmission is further supported, both by the injection challenge calibration experiment and by one of the vaccine trials in which unchallenged fish were cohabited (Fig. 3B and C). In the challenge calibration replication between tanks was poor (Fig. 3B), perhaps reflecting the low challenge doses and inoculum volume (50 μL). Nevertheless, just 10 cells by injection (9 CFU determined by viable count *post hoc*) was sufficient to kill fish in both replicate tanks, while 10^2^ CFU resulted in 50% mortality in both tanks and 10^3^ CFU resulted in 40% and 70% mortality in the replicate tanks (Fig. 3B). In contrast, all PBS injected fish remained healthy through the duration of the experiment and were feeding well at termination 21 days postchallenge (Fig. 3B). A similar prechallenge conducted with 10-fold higher doses by injection in larger fish (~78g), resulted in mortalities from 80 to 100% in injected fish, but no mortality occurred in cohabiting unchallenged fish (Fig. 3C).

In the vaccine trial cohabitation, fish were kept at a very high stocking density (~26 kg/m^3^) and 90% the stock (158 out of 176 fish) were injected with a high challenge dose of 10^5^ CFU per animal. The latter dose was indeed very high as it essentially eliminated all challenged fish, including vaccinates in 3 days (Fig. 3D). 17 out of 18 unchallenged cohabitants showed no behavioral signs of infection and had no visible organ damage upon postmortem examination and no bacteria in the caudal kidney when experiment was terminated after 19 days postchallenge. However, one unchallenged fish developed typical Pdp infection 6 days postchallenge (including darkening of the skin, enlarged liver, and Pdp granulomas in caudal kidney). This fish was mishandled (dropped) during transfer post-anesthesia and had a hematoma on its head.

In all experiments where unchallenged fish were cohabited with infected fish, using fish of different sizes and from different genetic stock, even at very high stocking density (>22 kg m^−3^ in all experiments) and with high challenge loads, infection was not transferred to uninfected fish in the same tanks. While direct immersion challenge did result in some mortality in small fish, these fish were subjected to a very high concentration of bacteria in a very small container. Since such conditions are not likely to be found outside experimental systems, it shows that although Pdp transmission through water is possible *per se*, it is highly unlikely to happen in healthy farmed or wild YTK. For example, in cage reared YTK in South Australia, a 44 m diameter sea pen would be stocked with around 100,000 20 to 30 g fingerlings, grown on to 1 to 1.5 kg, then split into two pens. A maximum pen biomass may occasionally reach 280,000 kg close to harvest (4 to 5 kg fish) in pens of ~13,500 m^3^, equivalent to about 20 kg m^−3^. For the majority of the farm cycle, however, density will never exceed 15 kg m^−3^, far below the densities in the cohabitation experiments described here.

### A novel model for Pdp pathogenicity.

**FIG 4:**
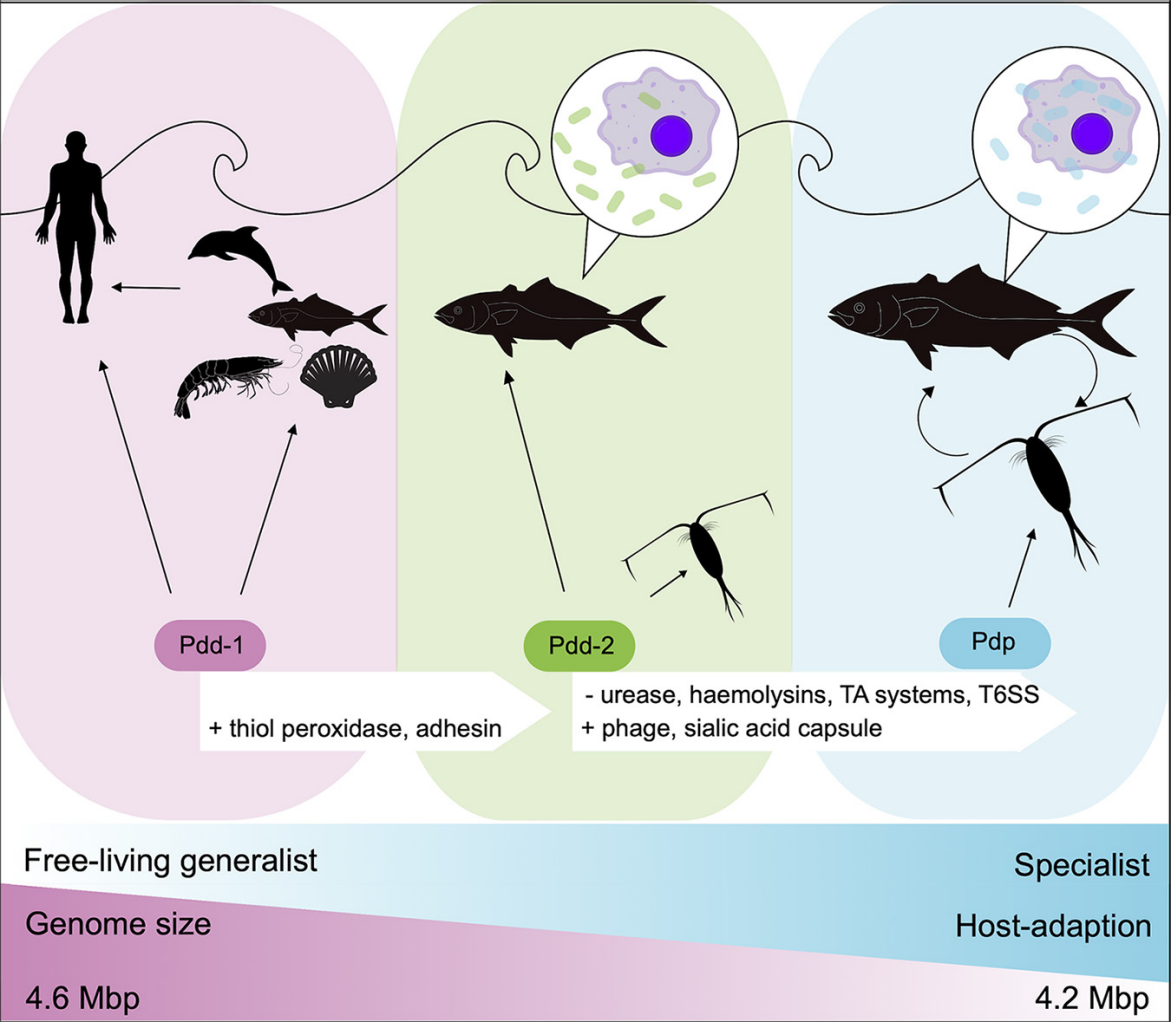
Evolutionary model of P. damselae subsp. *piscicida* and P. damselae subsp. damselae pathogenicity. Pdd-1 are a free-living, marine bacteria that cause opportunistic infections in immunocompromised animals, including humans that handle contaminated tools or water or infected animals. Acquisition of a thiol peroxidase and of the ShdA adhesin may have allowed Pdd-2 to occasionally enter the fish sentinel cells and colonize zooplankton species. The loss of urease seems to be the key change toward the emergence of Pdp. Lack of it may have led to a stable colonization and transmission from zooplankton to healthy fish. At the same time though, Pdp has acquired additional genes that allowed it to hide from (e.g., sialic acid capsule) and withstand (e.g., the stealth CR1 domain-containing protein) the fish immune system, and to successfully enter and replicate inside fish macrophages.

Pdp is a highly adapted fish primary pathogen infecting juvenile and adult YTK, but it is still not clear how it spreads among hosts. Transmission by immersion has been demonstrated in small juvenile turbot and eel (8 to 10 g) in marine and brackish water, respectively (66), albeit requiring very high doses 8 × 10^6^-1 × 10^8^ for 1 h at 22°C. The resulting infections did not display typical pathology associated with Pdp in larger animals (kidney and spleen granulomas, septicemia), rather fish developed infected skin lesions, although Pdp was isolated from internal organs in pure culture suggesting progression to systemic infection (67). Although Pdp shedding from infected fish has also been detected in experimental systems up to 29 days postinfection by injection of European sea bass (*D. labrax*) (68) it has been reported that it can survive in the water only for a short time (32, 69). Whether due to insufficient shedding, lack of survival in the water, lack of invasiveness through intact surface and gut layers, or a combination of these factors, we have established a lack of efficient transmission through water or direct contact in four separate cohorts of YTK of differing sizes held in RAS. Conversely, transmission by vector would facilitate the contact between nonmotile Pdp and its hosts and, consequently, colonization, and would allow Pdp to retain its extremely high virulence in fish (70). Our comparison between P. damselae subsp. damselae and P. damselae subsp. *piscicida* genomes has revealed several changes that together suggest a novel model for Pdp epidemiology. Similar to Y. pestis, loss of the urease gene may have represented the pivotal event that allowed Pdd-2 strains to initially colonize and survive within an arthropod vector. The toxicity to insects of ureases from plants and bacteria is widely reported, with toxicity attributed to both ureolytic and nonureolytic processes (71). For example, *Photorhabdus* sp. and *Xenorhabdus* sp., symbiotic in nematodes pathogenic to the fall armyworm *S. frugiperda*, produce urease that is detected in the insect’s hemolymph during infection preceding mortality (72). Further, the fitness of the insect pathogen Bacillus thuringiensis for its host, the gypsy moth, is highly correlated with urease production (73). In Y. pestis, transmission by the flea is directly attributed to loss of ureolytic activity *via* loss of function mutation in *ureD* (17). While there are no reports of urease toxicity in marine arthropods, the similarities in evolution of flea-borne Y. pestis from fecal-orally transmitted Y. pseudotuberculosis and the pathways we report for Pdp from Pdd are striking in their similarity. Furthermore, Pdp outbreaks have been reported concomitantly with infestations by the copepod *Caligus elongatus* in European sea bass *Dicentrarchus labrax* in Egypt (41) while kingfish farmed in cages in Western Australia are subject to frequent biting by gnathid isopods resulting in hematoma (Gavin Partridge, Department of Primary Industries and Rural Development Western Australia, Pers. communication.). Indeed, multiple genomic clues link Pdp to arthropods; the C-terminal domain of the unique AIP56 toxin displays high similarity with an uncharacterized hypothetical protein of the pea aphid (*Acrythosiphon pisum*) bacteriophage APSE-2 and with a hypothetical protein of *Danaus plexippus*, the monarch butterfly (74). Whether the vector scratches the protective mucosal layer of fish like *Argulus* sp. (75), feeds on blood meals like various copepod species (76–78) or parasitizes the fish such as the isopod *Cymothoa exigua* (79), the next step that leads to successful infection is adhesion to and invasion of the host. The Ig-like domain-containing protein with adhesin ShdA domain has been demonstrated to bind to fibronectin (80), a large glycoprotein present in vertebrate extracellular matrix and fluids, including blood plasma. This interaction may promote Pdp settlement and spread within the fish hosts. ShdA adhesins have already been reported to serve as entrance point for the viral hemorrhagic septicemia virus (VHSV) rhabdovirus in salmonids (81). Once Pdp has accessed the host, its two major virulence factors, AIP56 toxin and the siderophore piscibactin, promote its survival and replication by causing apoptosis in sentinel cells and scavenging iron from the host, respectively. Although critical for Pdp virulence, the *aip56* gene does not ‘define’ the subspecies as several early isolates lack it. Conversely, two other important genomic regions differentiate Pdd from Pdp: the partial AlpA phage and a conserved and distinct capsular operon, both involved in the immune evasion of Pdp from the fish immune system (Fig. 4).

### Conclusions.

Here, we have outlined how the genome of an opportunist, free-living bacterium Pdd has gradually become that of a highly adapted, host-restricted pathogen Pdp. Acquisition of a thiol peroxidase, adhesins, sialic acid capsule, and plasmids critical for virulence followed by loss of urease, Toxin-antitoxin systems, T6SS and hemolysins have refined the repertoire of specialist functions of Pdp. The loss of urease, a known toxin of arthropods (82), the reported co-isolation of Pdp from fish infected with copepod parasites (41), coupled with the lack of transmissibility through water reported in the present study are supportive of a vector-borne lifestyle for Pdp. These events share many similarities with the transition of the deadly Yersinia pestis from the less virulent Y. pseudotuberculosis, suggesting that common evolutionary mechanisms extend across both terrestrial and marine ecosystems.

## MATERIALS AND METHODS

### Genome assemblies.

DNA from Australian Pdp and Pdd strains (Table S1, Supplemental material) was extracted and sequenced, assembled and closed using Nanopore long-reads and Illumina NextSeq short reads as previously described (33). All the genomes available to date on NCBI were also retrieved (Table 1), their identities checked and excluded when uncertain. To determine whether assembly quality and sequencing technology impacted subsequent analyses, each of the draft assemblies was used to generate a simulated set of Illumina reads with InSilicoSeq v.1.5.0 (83), –model miseq, to obtain an ~50-fold coverage. Assemblies of Illumina reads was performed in SPAdes v.3.14.0 (84) with the flags –careful and Shovill v.1.1.0 (https://github.com/tseemann/shovill) with the flag –gsize 5m and –depth 0 to ensure the 50× coverage was maintained. The quality of the assemblies was then evaluated using the online version of QUAST (85), with SPAdes assemblies having generally a higher quality than the Shovill assemblies.

### Phylogenetic analysis.

Preliminary genome alignments were conducted with Parsnp (86) on assemblies and reassemblies both with and without an out-group (*P. profundum*). Predicted recombinant sites were identified and removed with Gubbins (87) and phylogenetic trees were constructed with RAxML (88) with bootstrap support of 1000 iterations in Geneious v.2020.1.2 with -m GTRGAMM and –asc-corr=felsenstein. Trees were visualized and rerooted in Dendroscope v.3.7.2 (89) and later annotated using Evolview v.3 (90). As no topological differences between phylogenies were found using the original and synthetic/SPAdes assemblies, the original assemblies of genomes as retrieved from NCBI were used in all subsequent analyses and new assemblies added as they became publicly available (Fig. S2 and S3 Supplemental material). To obtain a tree with genetic distances, complete and closed assemblies (9046-81, 91-197, Phdp Wu-1, QMA0505, QMA0506, QMA0365, QMA0366, QMA0508, QMA0509, QMA0510, QMA0511, QMA0512, QMA0513, CIP10276, KC-Na-1, KC-Na-NB1) were chosen. Parsnp was used to create a core genome alignment and putative recombinant sites removed with Gubbins. The phylogram was created in Geneious v.2020.1.2 using RAxML with the GTRCAT model, Felsenstein ascertainment correction and 1000 bootstrap support. Root-to-tip distance regression with isolation date was conducted with TempEst (91) to test whether the reconstructed phylogenetic tree showed any temporal signals. Isolates of P. damselae subsp. damselae are clustered in two separate groups Pdd-1 and Pdd-2. While the Pdd-1 isolates were isolated from a variety of hosts, including prawns, human, and fish, the Pdd-2 isolates were, with two exceptions from aquarium seawater (BT-6 and GCSL-P85), all isolated from aquatic animals. The most ancestral Pdd-2 strains, KC-Dl-1, KC-Na-1 and KC-Na-NB1, were isolated from cetaceans, but according to the description of their isolation (92, 93) the origin of KC-Na-1 and NC-Na-NB1 can be largely argued, as the animals were caught in nets as part of the bycatch and anal and blowhole swabs were taken, suggesting that Pdd may not have come from inside the mammals. For KC-Dl-1, no details about its isolation were found.

### Pangenome analysis.

Identification of sets of genes that were unique or intersecting the three major clades of P. damselae determined in the phylogeny was carried out as follows. First, Pirate v.1.0.4 (94) was used to obtain a pangenome excluding paralogs (–para-off) from the highest quality complete closed assemblies representative of the three clades identified with RAxML (QMA0508, QMA0512, QMA0513, Phdp Wu-1, 9046-81 and CIP102761 for Pdd-1; QMA0365, QMA0366, QMA509, QMA0510, QMA0511, KC-Na-NB1 and KC-Na-1 for Pdd-2; QMA0505, QMA0506, and 91–197 for Pdp). Next, groups of genes belonging only to Pdp and two phylogenetically distinct Pdd clades, and their intersections were estimated with a custom R script implemented in R v.4.0.4 (available at https://github.com/laurabaseggio/P.damselae.git), using the package VennDiagram v.1.6.20 (95) (https://CRAN.R-project.org/package=VennDiagram). Insertion sequence elements (ISEs) and transposases were excluded from the analysis. Finally, a BLAST search in Geneious v.2020.1.2 was performed to refine each group at a 95% coverage and 95% identity, including all strains. For each gene of interest, promoters were analyzed with BPROM (http://www.softberry.com/), while for the genes annotated as hypothetical proteins a conserved domain search was performed on the NCBI Conserved Domains Database.

### H_2_O_2_ resistance.

Two Pdp strains (QMA0505 and QMA0506) and two Pdd isolates from each cluster (QMA0508 and QMA0513 from cluster Pdd-1 and QMA0365 and QMA0509 from cluster Pdd-2) were grown overnight in TSB1 at 25°C. In a U-bottom 96-well plate 2-fold serial dilutions of H_2_O_2_ were made starting from 100 nM H_2_O_2_ in Tryptic Soy Broth supplemented with 1% NaCl (TSB1) to a final volume of 100 μL and inoculated with 5 μL containing 5 × 10^5^ cells. The plate was incubated in a BMG FLUOstar OPTIMA Microplate Reader at 25°C, 120 rpm agitation for 15 sec before each reading of OD (600 nm) performed every hour. The minimum bactericidal concentrations (MBC) for each strain were also calculated. In a U-bottom 96-well plate 2-fold dilutions of H_2_O_2_ were made starting from 300 nM H_2_O_2_ in TSB1 and inoculated with 5 × 10^5^ cells. The plate was incubated at 25°C without shaking. After 1h and 2h, 5 μL of culture from each well were spotted on a Tryptic Soy Agar supplemented with 1% NaCl (TSA1) plate, allowed to dry, and incubated overnight at 25°C. 48 h later, plates were examined for the presence of visually detectable growth.

### Assessment of Pdp transmission through water and direct contact.

**(i) Challenge trial context.** As part of ongoing commercial fully blind and repeated trials for registration of a vaccine, Pdp challenge models in Yellowtail Kingfish (*Seriola lalandi*, YTK) were conducted using a tag and mix approach during the development of the model and subsequent candidate trials between 2018 and 2021. In general, batches of YTK were air-freighted direct from hatcheries at Arnault Bay, South Australia or Fremantle, Western Australia as 1 g fry. Fish (100 per box) were packed in 8 L seawater, double-contained in plastic bags inflated with pure oxygen and sealed into standard Styrofoam fish boxes. On arrival at the University of Queensland (UQ), fish were acclimatized in natural filtered (200 μm) seawater in 1000 L circular tanks (800 L fill volume) in an 8 ton recirculating aquaculture system (RAS). The RAS was equipped with a gravity-fed 38 μm drum screen filter and 800 L settlement sump for particle filtration, a 750 L protein skimmer, an 800 L biofilter containing ~200 L Kaldnes K1 medium with vigorous aeration and 700 L clean sump. Seawater was conditioned to 23 ± 1°C from the clean sump using a 3 hp heat exchanger and cycled through a 150 W UV sterilizer before being returned to growth tanks at a rate of 3 to 4 exchanges per hour. Water quality was monitored daily for DO% saturation, pH, temperature, conductivity, total ammonia nitrogen, nitrite, and nitrate. Approximately 10% total RAS volume was replaced each day with clean filtered seawater from storage tanks to maintain nitrate levels, and the pH was adjusted to 8.2 ± 1 with 5 M NaOH. Photoperiod was maintained at 13:11h light:dark via LED arrays with full daylight spectrum. Fish were fed commercial extruded diets appropriate to the size of fish (Pelagica Hatchery, Ridley Aqua Feeds, Narangba) twice per day. Fish were reared until 15 to 25 g and then all fish in the cohort were tagged under general anesthetic (Aqui-S, Primo Aquaculture, Australia) with 8 mm RFID microchips (SwissPlusID, Australia) by injection into the dorsal muscle immediately distal to the head. Fish were then allowed to recover from tagging for 2 to 3 weeks and fed at 2.5% body weight once per day at 22°C. This permitted complete recovery and wound healing from the tagging process indicated by the disappearance of tagging-related hematomas. For vaccine trials, fish were scanned, the barcode recorded and vaccinated in groups by intraperitoneal injection (50 μL) under general anesthetic with equal numbers of each cohort of vaccinated and control animals mixed within each holding tank, then reared for 800 to 900°days (time in days x temperature in °C to account for ectothermic physiology) postvaccination prior to challenges. 3 weeks prior to scheduled challenge of the vaccination experiments, pilot prechallenge pathogen dose titrations were conducted in tagged fish from the same cohort. While the numbers of fish required for each experiment were determined carefully and ordered, farmers always shipped excess animals to account for loss during transport and acclimatization. In practice, losses were very low (0 to 1%) and therefore excess fish were tagged. Consequently, during all pathogen dose titration prechallenges and during some of the vaccine trials, cohorts of noninfected (sterile PBS-injected) fish were included as cohabitants. We detail some of these trials below in different cohorts of fish at different sizes as they provide interesting observations on the infectivity of Pdp.

**(ii) Infection model/delivery route comparison in small fish (injection versus immersion versus immersion/wound).** Lethality of Pdp isolate QMA0506 (33) was assessed in 10 g YTK following delivery by intraperitoneal (IP) injection, by immersion, and by immersion in wounded fish in a 4 ton experimental RAS. The smaller scale RAS (generally used for feeding trials) comprises 20 × 130 L (100 L fill volume) growth tanks, 4 × 50 μm bag filters, 2 × 400 L biofilters containing approximately 120 L aerated Kaldnes K1 medium, 2 × 25 L protein skimmers, 2 × 300 L clean sumps each fitted with 3 KW chiller units and 600 W titanium heaters. Water quality was measured and adjusted/exchanged as described for the larger system (above). Fish were handled under general anesthetic (Aqui-S, Primo Aquaculture, Australia). For the IP challenge 50 μL of bacterial suspensions were injected at the concentrations of 5 × 10^2^, 5 × 10^3^, 5 × 10^4^, 5 × 10^5^, 5 × 10^6^ CFU per fish. For the second and third methods, fish and wounded fish (the wounding consisted of a 1 to 2 mm cut on the tail of anesthetized animals with a scalpel blade) were immersed for 2 min in 5 L of bacterial suspensions at concentrations of 10^4^, 10^5^, 10^6^ and 10^7^ CFU/mL. For each treatment and dose, 20 fish were challenged, marked with saturated Alcian blue using a Panjet and separated evenly into two replicate 100 L tanks, 10 per tank. The final numbers of challenged fish per tank were 50 for IP injection, 60 in immersion and 60 in wounded immersion equivalent to a stocking density of 5 to 6 kgm^−3^. Moribund fish (dark, erratic swimming, separated from the school), were euthanized (overdose of Aqui-S), and time, tank and treatment were recorded.

**(iii) Prechallenge pathogen dose titration experiments.** YTK (Q4.1 ~78 g, Q5.1 ~55g) tagged with 8 mm RFID microchips (above) were challenged under general anesthetic by IP injection with 10^4^, 10^3^ and 10^2^ (Q4.1) or 10^3^, 10^2^ and 10 CFU/fish (Q5.1) in 50 μL sterile PBS or injected with 50 μL sterile PBS as a handling control and 10 fish per treatment were cohabited in two replicate 100 L tanks in a 4 ton marine RAS. This resulted in each replicate tank containing 40 fish, equivalent to stocking densities of 31 and 22 kgm^−3^ in calibration trials Q4.1 and Q5.1, respectively. Fish were held at 22°C and fed at 2.5% BW once daily. Fish were monitored continuously and moribund fish (dark, erratic swimming, separated from the school) were removed, euthanized (overdose of Aqui-S), time and tank recorded and frozen pending subsequent identification by tag and bacteriological sampling.

**(iv) Cohabitation experiment during vaccine trial.** Tagged YTK (158× ~120 g), were challenged with Pdp isolate QMA0506 (10^5^ CFU in 100 μL of PBS) delivered per fish by IP injection, while 18 unchallenged fish received 100 μL of PBS. Fish were cohabited in a 1 ton (800 L fill volume) circular tank in the 8 ton marine RAS for 19 days, equivalent to a stocking density of 26 kgm^−3^. Fish were monitored continuously and moribund fish (dark, erratic swimming, separated from the school) were removed, euthanized (overdose of Aqui-S), time and tank recorded and frozen pending subsequent identification by tag and bacteriological sampling.

To confirm the cause of morbidity and identify the presence of potential subclinical infection in survivors in the above-described experiments, bacteriological samples from caudal kidney were taken with 1 μL loop and streaked on TSA/5% sheep blood. Identity of isolates was confirmed by Pdp-specific PCR with 76a/b primer set (96). Survival analysis was performed using log-rank (Mantel-Cox) tests in GraphPad Prism v.9.1.2 for macOS.

**(v) Ethical statement.** All animal experiments were conducted under approval of the University of Queensland Animal Ethics Committee, Approval number 2019/AE000253 A trivalent vaccine for yellowtail kingfish.
