# Supplementary material for: The Evolution of a Specialized, Highly Virulent Fish Pathogen through Gene Loss and Acquisition of Host-Specific Survival Mechanisms
Source: Appl Environ Microbiol. 2022 Jul 11;88(14):e00222-22. doi: 10.1128/aem.00222-22 (PMC9317898; doi:10.1128/aem.00222-22)
Supplement: Supplemental file 1 — Table S1 and Fig. S1 to S7. Download aem.00222-22-s0001.pdf, PDF file, 6.8 MB [file aem.00222-22-s0001.pdf]

The evolution of a specialised, highly virulent fish pathogen through gene loss and acquisition of host-specific survival mechanisms

Laura Baseggio, Oleksandra Silayeva, Jan Engelstädter and Andrew C. Barnes

The University of Queensland, School of Biological Sciences, Brisbane, QLD 4072, Australia

Corresponding author

a.barnes@uq.edu.au

Supplementary Material

Supplementary Table S1. Australian *P. damselae* subsp. *damselae* isolates sequenced in the present study. All strains were isolated from *Seriola lalandi*.

Strain	Date of isolation	State	GenBank accession number
QMA0365	2010	South Australia	CP090501-CP090503
QMA0366	2010	South Australia	CP090498-CP090500
QMA0508	2015	South Australia	CP090495-CP090497
QMA0509	2015	Western Australia	CP090493-CP090494
QMA0510	2015	Western Australia	CP090489-CP090492
QMA0511	2015	Western Australia	CP090487-CP090488
QMA0512	2016	Western Australia	CP090485-CP090486
QMA0513	2016	Western Australia	CP065041-CP065043

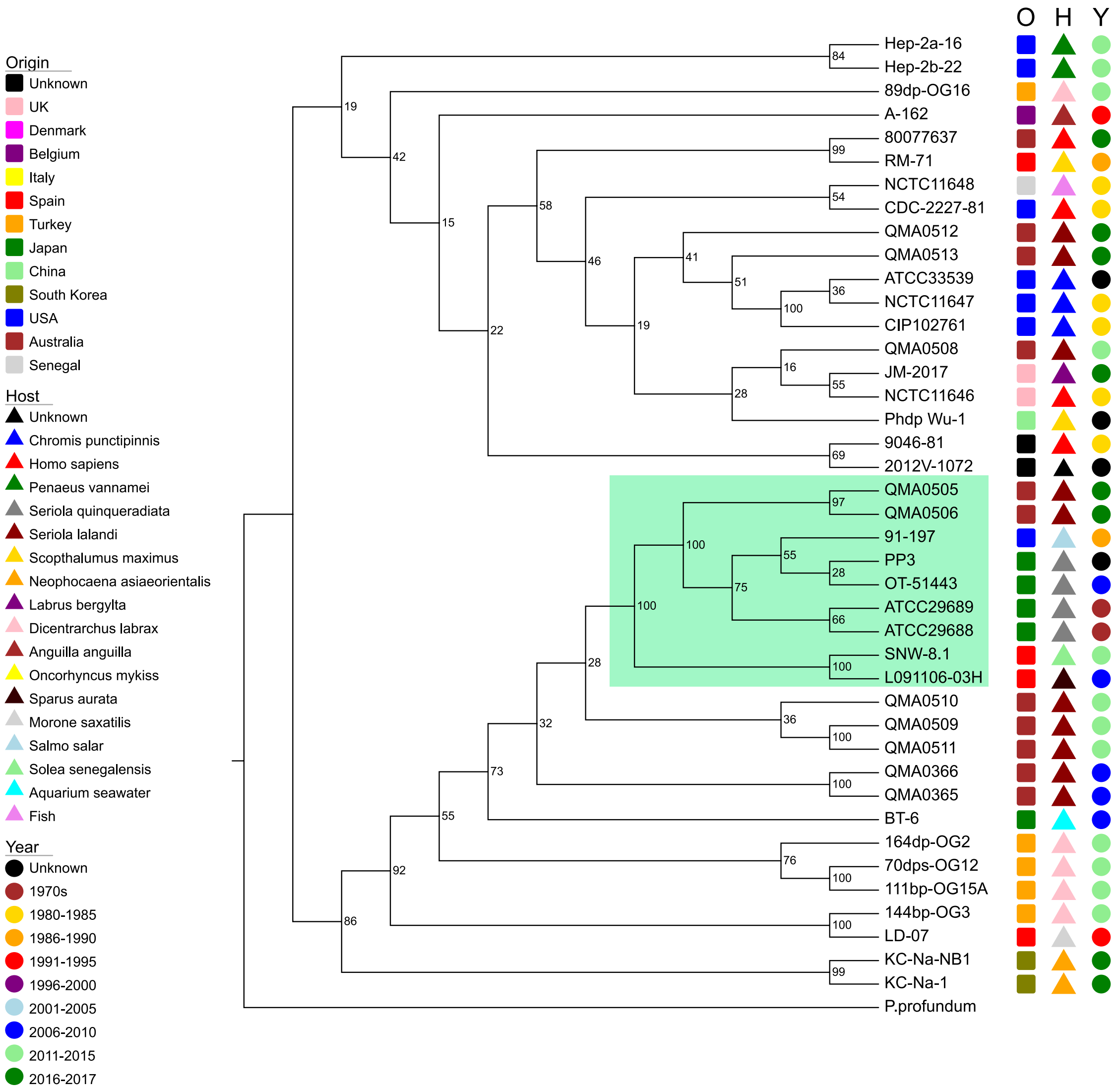

Supplementary Figure S1. Cladogram prepared with unmodified NCBI assemblies of *Photobacterium damselae* genomes. *Photobacterium profundum* as outgroup, Pdp strains are highlighted in light green

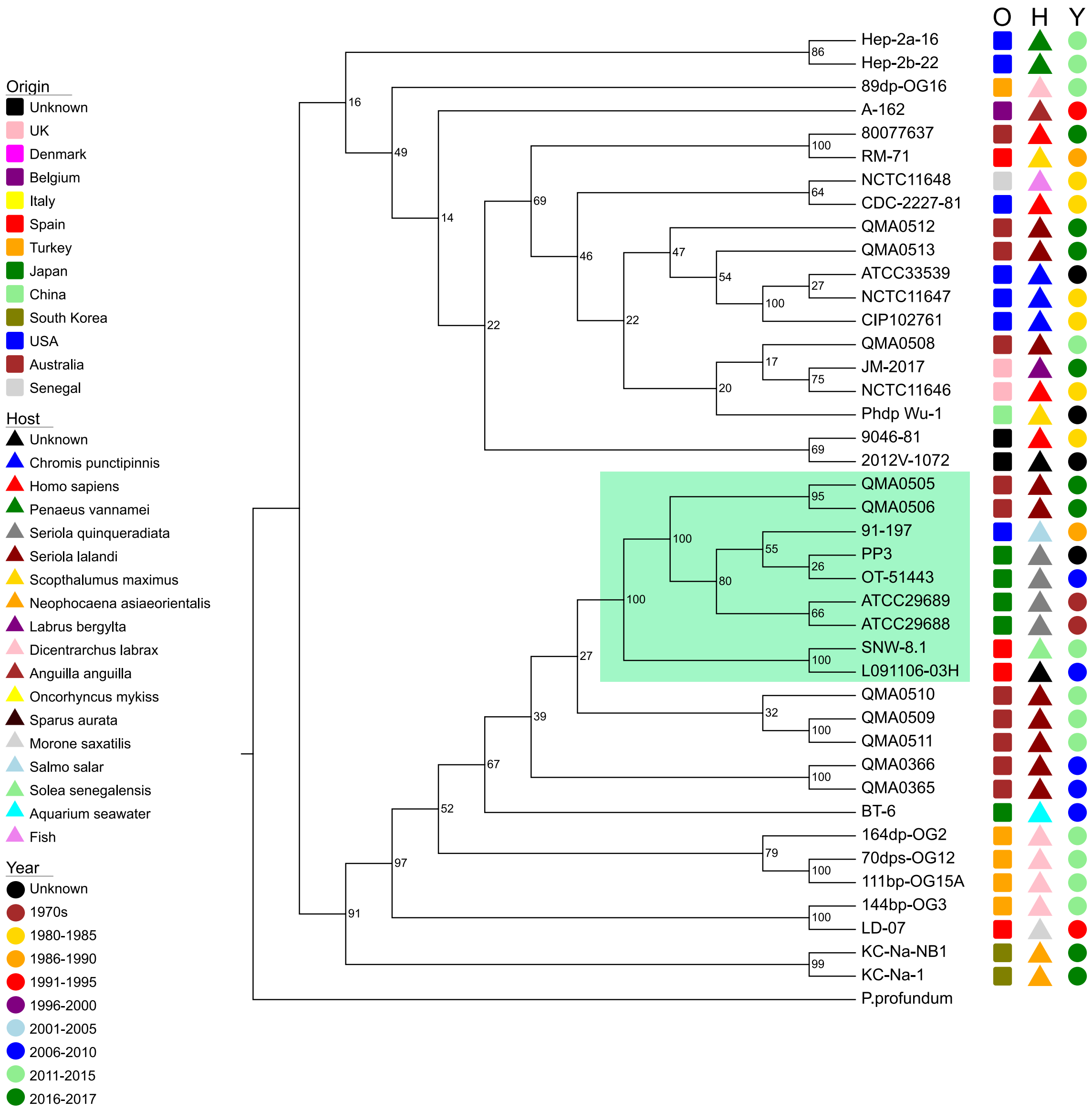

Supplementary Figure S2. Cladogram prepared with re-assembled of *Photobacterium damselae* genomes. *Photobacterium profundum* as outgroup, Pdp strains are highlighted in light green.

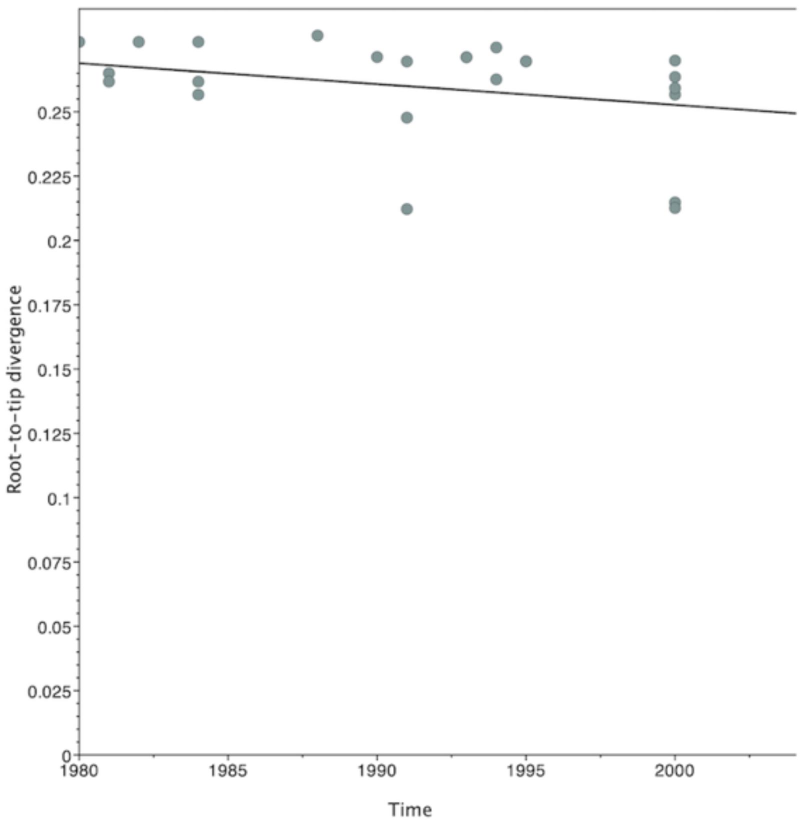

Supplementary Figure S3. ***P. damselae*** **phylogenetic branch length root-to-tip regression against time.** Regression was performed with TempEst on distances estimated by maximum likelihood using best root option. Date of isolation to present was taken as a proxy for time.

A

B

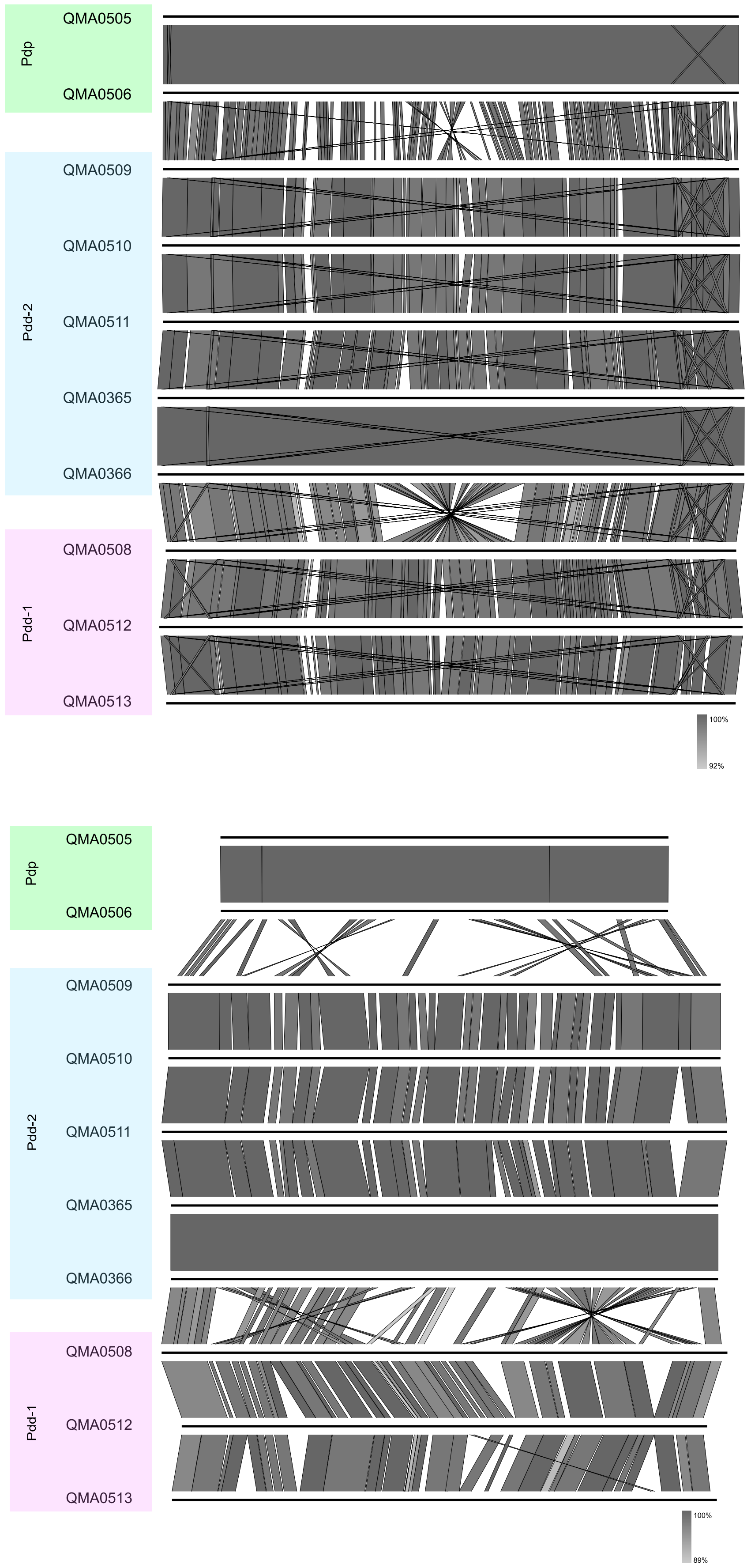

Supplementary Figure S4. Comparison of A) chromosome 1 and B) chromosome 2 of *Photobacterium damselae* isolates from Australia using EasyFig.

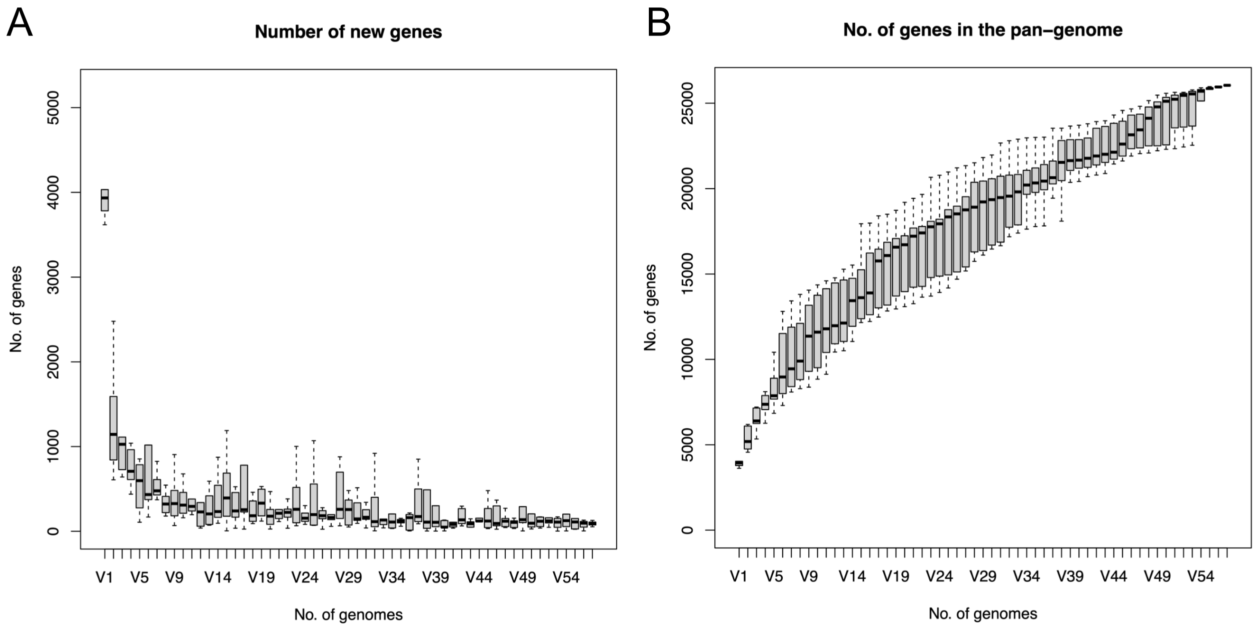

Supplementary Figure S5. ***Photobacterium damselae* pangenome.** Cumulative plots of the number of new genes (A) and number of genes (B) in *P. damselae* pangenome based on 10 random seed genomes.

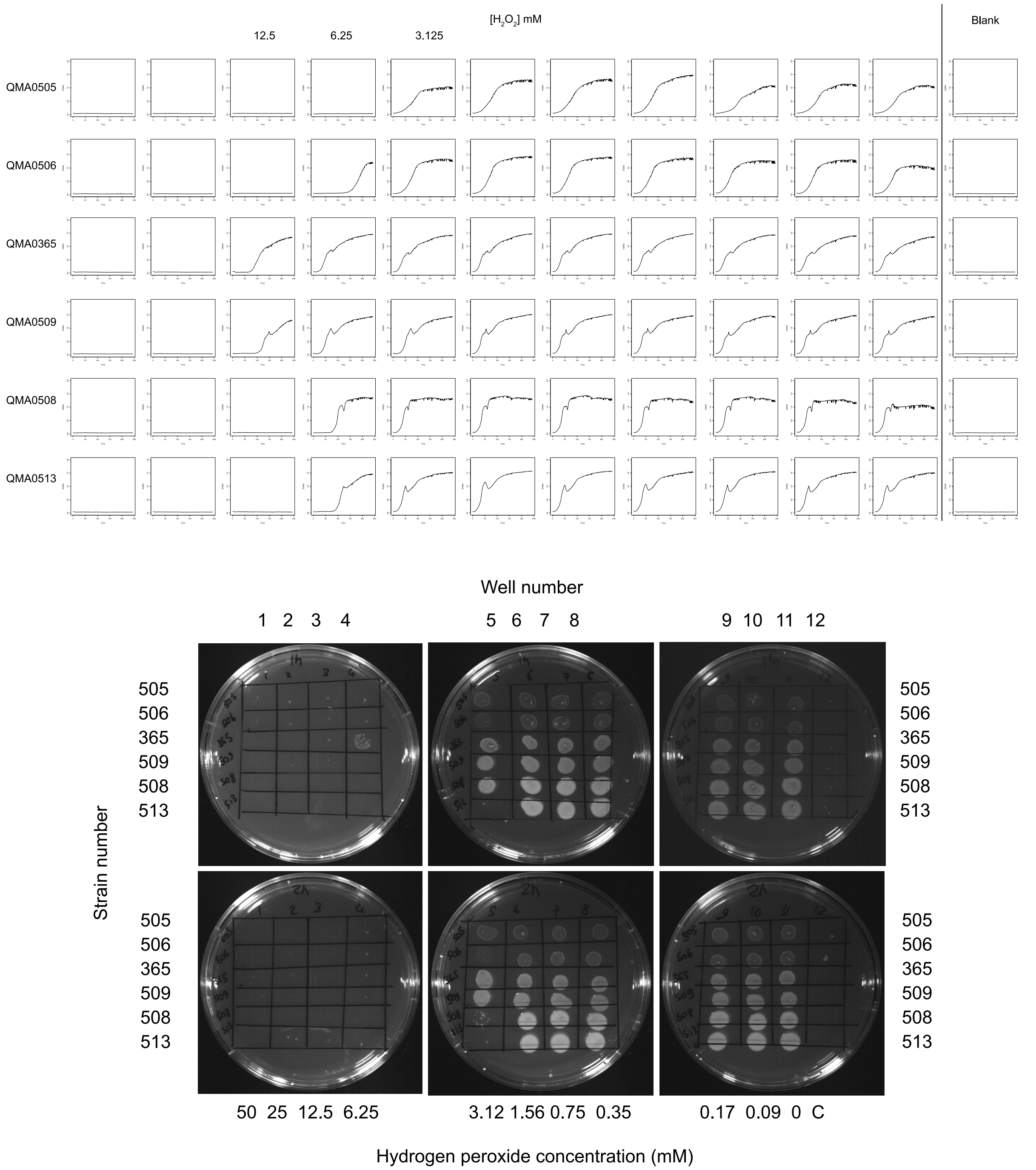

Supplementary Figure S6. Phenotypic traits of *P. damselae* subspecies *damselae* from Clades 1 and 2 and *P. damselae* subspecies *piscicida.* A) Growth curves of QMA0505 (Pdp), QMA0506 (Pdp), QMA0365 (Pdd-2), QMA0509 (Pdd-2), QMA0508 (Pdd-1) and QMA0513 (Pdd-1) in presence of decreasing concentration of H_2O_2. B) Minimum bactericidal concentration (MBC) of H_2O_2 for *P. damselae* strains. The concentration of H_2O_2 decreases from 150 mM in 1 to 0.146484375 mM in 11, column 12 being the negative control (TSB-1 only). Cells were incubated for 1 or 2 hours in the top and bottom panel respectively. Each row represents one of the strains in the following order: QMA0505 (Pdp), QMA0506 (Pdp), QMA0365 (Pdd-2), QMA0509 (Pdd-2), QMA0508 (Pdd-1) and QMA0513 (Pdd-1).

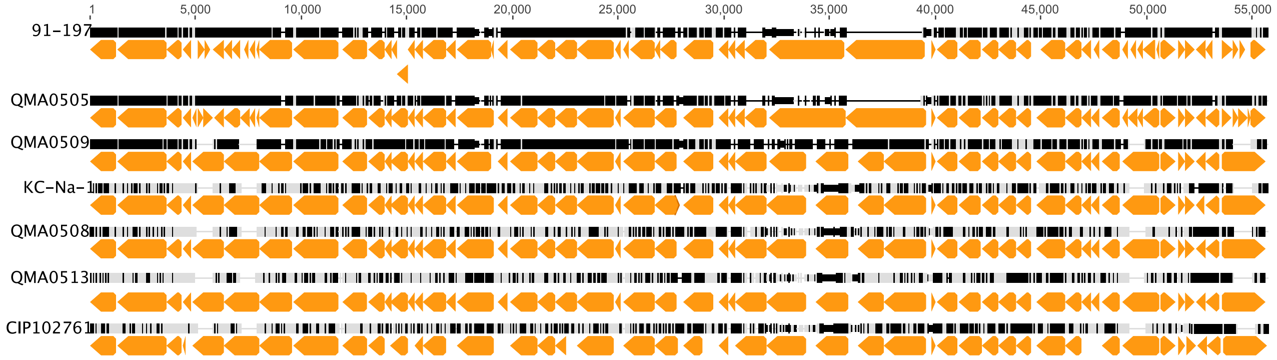

Supplementary Figure S7. Alignment of the flagellar operon. Diagram includes genomic region from two Pdp strains (91-197 and QMA0505), two Pdd strains from the Pdd-2 cluster (QMA509 and KC-Na-1) and two Pdd strains from the Pdd-1 cluster (QMA513 and CIP102761).
